# Mechanical Behavior and Deformation Mechanisms of Nanotwinned Heterogeneous Ultrafine-Grained Austenitic Stainless Steel at Elevated Temperature

**DOI:** 10.3390/ma19132857

**Published:** 2026-07-04

**Authors:** Hongjing Ma, Rui Ke, Hua Zheng, Shuangqi Hu

**Affiliations:** 1 School of Nuclear Technology and Chemistry & Biology, Hubei University of Science and Technology, Xianning 437100, China; 2 Hubei Key Laboratory of Radiation Chemistry and Functional Materials, Hubei University of Science and Technology, Xianning 437100, China; 3 Collaborative Innovation Center for Advanced Steels, Wuhan University of Science and Technology, Wuhan 430081, China

**Keywords:** heterogeneous microstructure, elevated-temperature tension, deformation mechanism, detwinning

## Abstract

**Highlights:**

- Heterogeneous TW-UFG achieves high strength and ductility via HDI stress at room temperature.
- At 600 °C, homogeneous UFG deforms by dynamic recovery; TW-UFG involves coupled detwinning and dynamic recovery.
- At 10^−4^ s^−1^, dynamic recovery and detwinning raise the work hardening rate and enhance elongation of TW-UFG samples.

**Abstract:**

This study aims to investigate the effects of heterogeneous microstructure and strain rate on the microstructural evolution and mechanical properties of ultrafine-grained (UFG) austenitic stainless steel during elevated-temperature tension. In this research, 17Cr-10Ni austenitic stainless steel was rolled to a 60% reduction in thickness at room temperature and 200 °C, followed by annealing at 1000 °C and 500 °C, respectively. The microstructural evolution of the annealed samples and high-temperature tensile specimens was characterized using optical microscopy, transmission electron microscopy, scanning electron microscopy equipped with electron backscatter diffraction, and X-ray diffraction. Results show that at room temperature, the heterogeneous twinned UFG (TW-UFG) sample, influenced by hetero-deformation-induced stress strengthening, maintains good ductility while exhibiting higher strength than the uniform UFG sample. During tensile deformation at 600 °C, grain refinement still contributes to strengthening, and the dominant deformation mechanism in the uniform UFG sample is dislocation dynamic recovery, whereas in the TW-UFG sample is detwinning combined with dynamic dislocation recovery. At low strain rates (10^−4^ s^−1^), sufficient dynamic recovery and detwinning in the TW-UFG sample delay plastic instability and improve elongation.

## 1. Introduction

Austenitic stainless steels (ASSs), owing to their outstanding corrosion resistance, workability, and comprehensive mechanical properties, hold substantial value in high-temperature service applications, including nuclear power, aerospace, and energy systems. However, traditional coarse-grained ASSs exhibit relatively low yield strength at room temperature [1]. They are susceptible to dynamic recovery, recrystallization, and grain boundary sliding at elevated temperatures, leading to significant strength softening and plasticity degradation, thereby making them unable to meet the strict requirements of advanced equipment for high-temperature load-bearing capacity, creep resistance, and structural stability [2,3,4].

Studies have demonstrated that grain refinement increases grain boundary density and impedes dislocation motion at elevated temperatures, thereby enhancing high-temperature mechanical properties. Lei et al. [5] found that grain refinement significantly increases the yield strength of austenitic stainless steel at room temperature. Nevertheless, as the tensile temperature increases from room temperature to 100 °C, the stacking fault energy (SFE) rises to 38 mJ/m^2^, causing the deformation mechanism to transition from transformation-induced plasticity (TRIP) and twinning-induced plasticity (TWIP) to slip via stacking faults and planar slip, which leads to little change in the yield strength of nano- or ultrafine-grained austenitic stainless steels, yet a substantial reduction in work hardening capacity along with a decrease in tensile strength and elongation. Xu et al. [6] showed that high-density grain boundaries in fine-grained samples effectively impede dislocation motion, enabling them to maintain higher tensile strength than coarse-grained samples, even at 600 °C. However, as the temperature further increases, dynamic recrystallization softening occurs during deformation, weakening the strengthening effect of grain boundaries. Huang et al. [7], through dislocation dynamics simulations, showed that the influence of dislocation climb on the high-temperature mechanical behavior of fine-grained polycrystalline aluminum is more pronounced when the grain size reaches a critical value (~0.5 μm), reducing the grain-refinement strengthening effect. In summary, increased temperature intensifies the thermal motion of atoms in metals, accelerates dislocation annihilation and promotes grain boundary sliding, as a result weakening or even completely negating the strengthening effect of grain boundaries.

In recent years, employing grain refinement coupled with heterogeneous structure design has been used to solve the issue of elongation degradation caused by fine-grain strengthening, making it an efficient strategy for achieving a balance between strength and toughness in metallic materials. The excellent mechanical properties are attributed to the hetero-deformation-induced (HDI) strengthening and hardening effect. Under loading, the soft-phase region undergoes preferential deformation, while the hard-phase region deforms less and generates a strain gradient, which promotes the accumulation of geometrically necessary dislocations (GNDs) near the heterogeneous interface. The resulting long-range internal stress field including the backward stress in the soft region and the forward stress in the hard region jointly constitutes the heterogeneous deformation-induced stress, which not only enhances the yield strength but also maintains a high processing hardening rate during the deformation process [8]. Among various heterostructures, nanotwinned ASSs represent a particularly typical and effective configuration, as nanotwin bundles serve as hard domains embedded in a softer recrystallized matrix, providing both strengthening and sustained work hardening capacity [9,10]. Compared with conventional high-angle grain boundaries (HAGBs), twin boundaries exhibit superior thermal stability owing to their lower excess energy and coherent structure [11,12,13]. The study shows that deformation twins in 310S ASS exhibit good thermal stability below approximately 800 °C, whereas recrystallization occurs along twin boundaries above 800 °C [14]. In a twinned-strengthened Fe-30Ni-15Cr austenitic steel developed by Liu et al. [15], the synergistic influence of nanoscale twin boundaries and coherent secondary phases at interfaces enables the material to maintain a yield strength of 780 MPa even at 700 °C. This combination of strengthening capability and thermal stability makes nanotwinned domains particularly suitable as the hard constituent in heterostructures designed for elevated-temperature applications. Despite these promising attributes, the high-temperature deformation behavior of heterostructures containing nanotwinned domains has received limited attention. Most studies on heterostructured ASSs have focused on room-temperature mechanical properties, while the coupled effect of twin-induced heterogeneity on high-temperature tensile response has received even less investigation.

In this study, tensile tests were conducted on two types of UFG microstructures in 17Cr-10Ni ASS, which were processed to have similar average grain sizes but different architectural uniformity: a homogeneous UFG structure obtained by cold rolling and recrystallization annealing and a heterogeneous TW-UFG structure obtained by warm rolling and subcritical annealing. The comparable grain sizes allow any differences in mechanical behavior to be attributed to the presence or absence of twin-induced heterogeneity rather than to grain size effects. Specific focus will be devoted to revealing the dominant deformation and softening mechanisms operative in each microstructure at 600 °C and to clarify how twin-induced heterogeneity governs the elevated-temperature behavior of ASSs.

## 2. Materials and Methods

### 2.1. Materials and Preparation Processes

**Figure 1:**
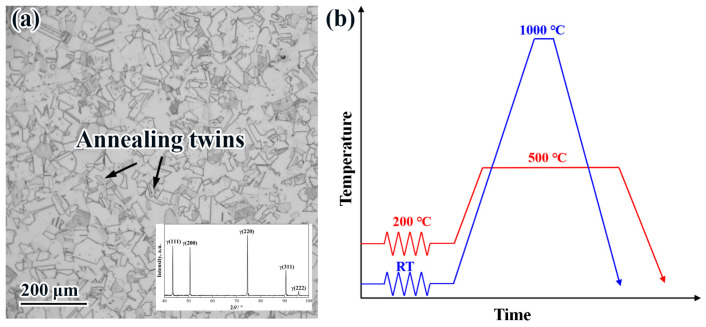
(**a**) Optical micrograph of the as-received sample and corresponding XRD pattern; (**b**) schematic diagrams of different heat treatment processes.

The experimental material was a 3.0 mm thick, solution-annealed 17Cr–10Ni ASS, for which microstructure and X-ray diffraction (XRD) patterns are presented in Figure 1a. The average grain size was measured to be 12.2 ± 8.3 μm, and its nominal chemical composition is provided in Table 1.

A multifunctional rolling mill (YZ-2, Yunnan Metallurgy Kunming Heavy Industry Co., Ltd., Kunming, China)was used to perform multiple passes of rolling on the original sample at both room temperature (20 °C) and 200 °C, with strict control of temperature changes during rolling. The total reduction in thickness (reduction thickness/original thickness × 100%) was set at 60%. The cold-rolled sheet was then annealed at 1000 °C for 60 s to obtain the UFG sample; meanwhile, the warm-rolled sheet was subjected to subcritical annealing at 500 °C for 8 h to produce the TW-UFG sample. The schematic diagram of the heat treatment process is shown in Figure 1b.

### 2.2. Test Method

Dog-bone-shaped tensile specimens were machined along the rolling direction using a wire-cutting machine (TP745, Fangzheng CNC, Taizhou, China) from both annealed samples. The gauge length of the room-temperature tensile specimen was 25 mm, with a parallel section length of 35 mm and a width of 10 mm. Quasi-static uniaxial tensile tests were conducted on an AG Xplus 50 kN electronic universal testing machine (Shimadzu, Kyoto, Japan) with a strain rate of 10^−3^ s^−1^ during loading, and each set of specimens underwent three repeated tensile tests. Back stresses generated during deformation in UFG samples with different microstructures were measured through loading–unloading–reloading (LUR) tensile experiments. Displacement control mode was used during the tensile process, with a loading strain rate of 10^−3^ s^−1^ and an unloading rate of 2.5 × 10^−4^ s^−1^; reloading was initiated after stress was reduced to 50 MPa. Unloading tests were performed when the engineering strain reached 0.5%, 1%, 2%, 3%, 4.5%, 6%, 7.5%, 10%, 12.5%, and 15%. For high-temperature tensile testing, specimens were machined along the rolling direction, with a parallel section length of 30 mm and a width of 5 mm. Tensile tests were carried out at 600 °C with strain rates of 10^−3^ s^−1^ and 10^−4^ s^−1^, respectively, and each set of specimens was tested three times to obtain average mechanical properties.

After step-by-step grinding and mechanical polishing, the metallographic specimen was electrochemically etched using a 10% oxalic acid aqueous solution as the electrolyte at a current density of 1 A/cm^2^. After etching, the metallographic specimens were cleaned with anhydrous ethanol, blow-dried, and observed with an optical microscope (OM, Axioplan2 Imaging Zeiss, Jena, Germany). For electron backscatter diffraction (EBSD) specimens, the observation surface was the RD-TD plane, and the evolution of microstructure, grain distribution, and orientation of the as-received and rolled-annealed samples during tensile deformation was observed and analyzed, with an accelerating voltage of 20 kV and a step size of 100 nm. At least 500 grains were examined for each sample to ensure the reliability of the statistical data. The GND density was calculated from electron backscatter diffraction (EBSD) data using the Nye tensor approach implemented in the MTEX-6.1.0 toolbox for MATLAB R2023b. For the austenitic stainless steel with an FCC structure, the Burgers vector magnitude was taken as *b* = $a_{0}/\sqrt{2}$, (~0.254 nm). The active slip systems for the FCC crystal structure were defined as the {111}<110> slip systems [16].

After mechanical grinding of the test surface of the specimens, electropolishing was carried out, with a 10% perchloric acid ethanol solution as the electrolyte, stainless steel as the cathode, a polishing voltage of 20–25 V, and a polishing duration of 20–30 s. Channel 5 v5.12 analysis software was used to process the raw data. An X-ray diffractometer (XRD, Shimadzu, Kyoto, 6100, Japan) was employed to characterize the phases in the rolled and annealed samples and the tensile fracture region, with a Cu anode target (Cu-Kα radiation, wavelength λ = 1.5418 Å), a scanning range of 30–100°, and a scanning rate of 1°/min. The instrumental broadening was determined and corrected using a standard SiO_2_ powder measured under the same experimental conditions. For quantitative determination of the total dislocation density of samples, the XRD data were analyzed by the Rietveld refinement method using the HighScore Plus 3.0 software and calculated by the modified Williamson–Hall method [17], as shown in the following formula:

(1) $$\Delta K=\frac{0.9}{d}+{\left(\frac{\pi M^{2}b^{2}}{2}\right)}^{\frac{1}{2}}\rho^{\frac{1}{2}}\left(K{\bar{C}}^{\frac{1}{2}}\right)+O\left(K^{2}\bar{C}\right)$$

where *K* = 2sin*θ*/*λ*, *θ* is the Bragg diffraction angle, *λ* is the X-ray wavelength, and Δ*K* = 2cos*θ*(Δ*θ*)/*λ* is the physical broadening in reciprocal space. *M* is a constant determined by the outer cutoff radius of the dislocation, *b* = $a_{0}/\sqrt{2}$ is the Burgers vector, where *a*_0_ is the lattice parameter determined from the Rietveld refinement, ρ is the total dislocation density, *O* is a higher-order terms and $\bar{C}$ is the average dislocation contrast factor.

A transmission electron microscope (TEM, JEM-2100, JEOL, Tokyo, Japan) was used to observe the microstructure morphology of the specimens after tensile fracture, with an accelerating voltage of 200 kV during the test. The specimens were processed into 3 mm circular thin foils, followed by twin-jet electropolishing for thinning. The twin-jet electrolyte was a mixed solution configured with perchloric acid and anhydrous ethanol at a volume ratio of 9:1; the twin-jet temperature was controlled at −20 °C, and the twin-jet voltage was set to 30 V.

To characterize the fracture behavior, the fracture morphology of the tensile-tested specimens was characterized using scanning electron microscopy (SEM, VEGA3, TESCAN, Brno, Czech Republic). The fracture surfaces were observed in secondary electron (SE) mode at an accelerating voltage of 20 kV.

## 3. Results

### 3.1. Microstructure Analysis of Ultrafine-Grained Austenitic Stainless Steel

**Figure 2:**
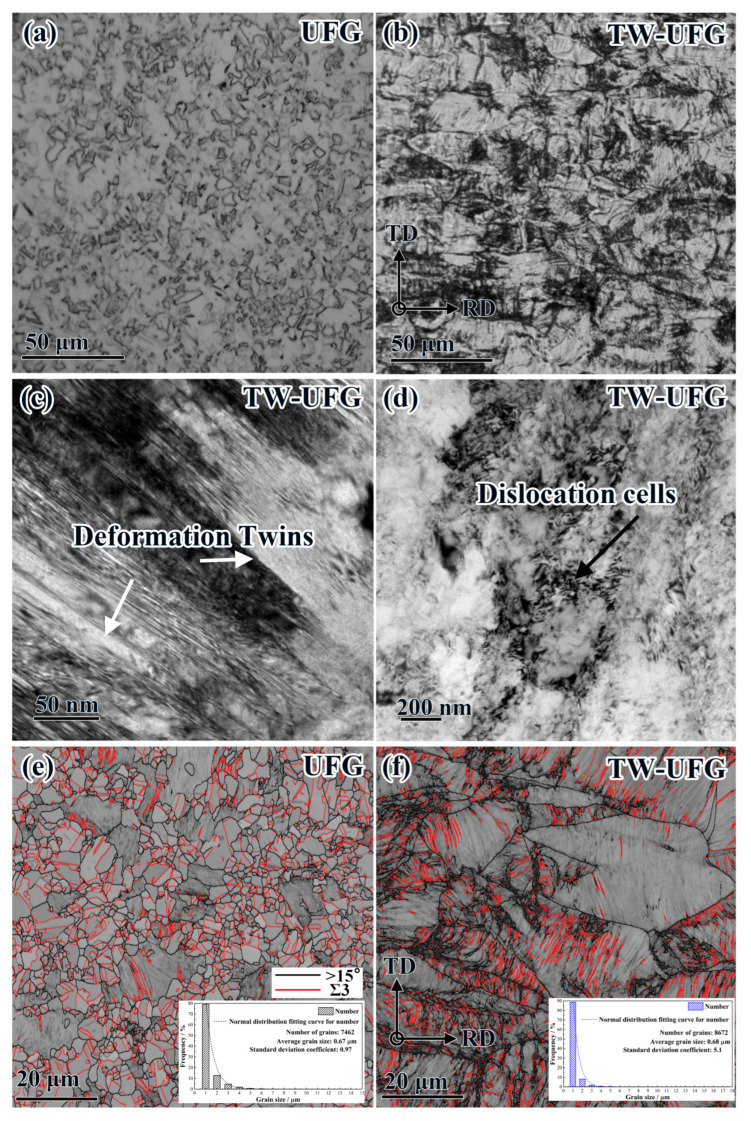
Metallographic images of (**a**) UFG and (**b**) TW-UFG samples; (**c**,**d**) TEM image of TW-UFG sample, (**e**,**f**) EBSD grain boundary (GB) + image quality (IQ) maps and corresponding austenite grain size distribution (number frequency) in samples: (**e**) UFG and (**f**) TW-UFG samples, respectively. In (**e**,**f**), the black and red lines represent high-angle (misorientation angle > 15°) and ∑3 twin boundaries (misorientation of 60° about a <111> axis, and the tolerance of misorientation angle for twin boundaries is 5°), respectively.

Figure 2 shows the microstructural morphology of the UFG and TW-UFG samples. According to the metallographic image of the UFG sample (Figure 2a), after isothermal annealing at 1000 °C for 60 s, recovery and recrystallization have occurred. From the grain boundary (GB) + image quality (IQ) map (Figure 2e), it can be observed that the UFG specimen mainly consists of equiaxed recrystallized austenite grains and a small amount of unrecrystallized deformed austenite, with annealing twins present within the recrystallized grains. As observed in Figure 2b, the austenitic grains in TW-UFG were elongated along the rolling direction (RD), and the coarse grains were subdivided by slip lines. TEM images of the TW-UFG sample (Figure 2c,d) show nanoscale deformation twin bundles and, within the untransformed austenite grains, dislocation cells that partition the original grains into finer subgrains. Statistical analysis of grain size was conducted using EBSD. The corresponding grain size distribution plots indicate that the UFG and TW-UFG samples have similar average grain sizes of 0.67 μm and 0.68 μm, respectively. Given that the thickness of individual deformation twin lamellae is below 20 nm, which is well beyond the spatial resolution limit of EBSD measurements, the twin boundaries detected by EBSD mainly reflect the interfaces of nanoscale twin bundles rather than individual twin lamellae. However, it is noteworthy that the coefficient of variation in grain size for the TW-UFG sample is significantly higher than that of the UFG sample (5.1 vs. 1.4), indicating that the presence of nanoscale deformation twin bundles not only reduces the grain size of the ASS but also creates a heterogeneous microstructure consisting of nanoscale twin bundles and micrometer-scale deformed austenite grains.

### 3.2. Mechanical Properties of Fine-Grained Strengthened ASS at Room Temperature

**Figure 3:**
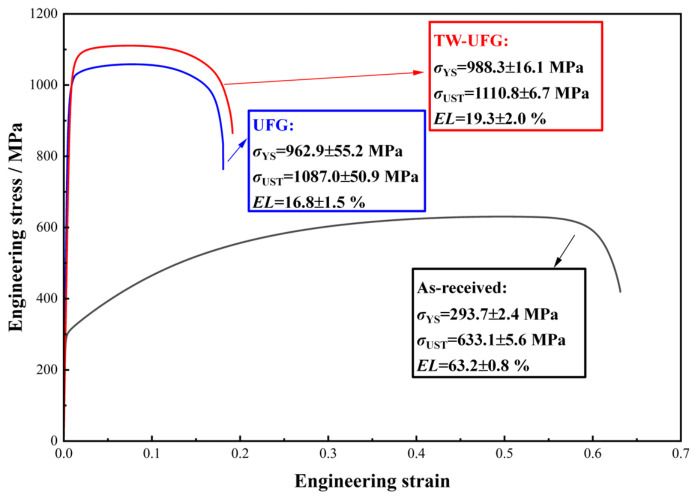
Engineering stress–strain curves of as-received, UFG and TW-UFG samples.

Figure 3 presents a comparison of the engineering stress–strain curves for the as-received 17Cr-10Ni ASS sample and the UFG and TW-UFG samples, and all samples display continuous yielding during tension. Consistent with previous literature reports, the strength of the steel increases notably as the grain size decreases. The yield strength of both the UFG and TW-UFG samples is more than three times greater than that of the as-received sample, whereas the ultimate tensile strength demonstrates a decreasing tendency. Nevertheless, the TW-UFG sample with a heterogeneous microstructure attains a strength–ductility product of 2.1 GPa·%, which is higher than that of the homogeneous UFG sample, suggesting a better balance between strength and ductility.

**Figure 4:**
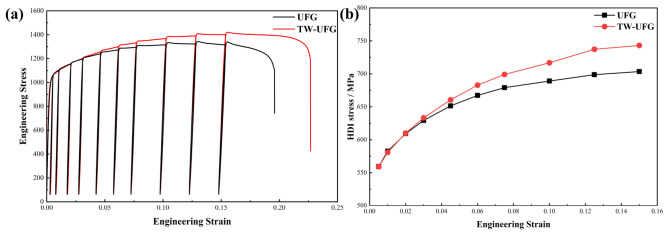
(**a**) LUR engineering stress–strain curves of UFG and TW-UFG steel and (**b**) evolution of HDI stress at different strains.

In recent years, researchers have investigated the mechanisms underlying the balance between high strength and plasticity in heterogeneous structural materials [18,19]. Hetero-deformation-induced (HDI) stress is one of the primary factors enabling this balance in such structures [20]. During plastic deformation, HDI stress is generated by the accumulation of geometrically necessary dislocations (GNDs) at heterogeneous interfaces, which simultaneously enhances both the strength and plasticity of materials by inducing work hardening and suppressing necking [21,22]. To evaluate the impact of HDI stress, LUR tensile tests were conducted on UFG steel and TW-UFG steel, with their LUR stress–strain curves shown in Figure 4a. During forward tensile loading, the macroscopic flow stress comprises the intrinsic lattice friction stress and the long-range back stress arising from geometrically necessary dislocation accumulation at hetero-interfaces. In the loading stage, the back stress acts opposite to the applied stress, serving as an internal resistance that elevates the flow stress threshold rather than counteracting the external stress. Only upon unloading does the back stress reverse its direction and align with the external stress; once its magnitude exceeds the lattice friction, it can drive reverse dislocation slip, manifesting as a distinct premature reverse yielding in the LUR hysteresis loops. This is the microscopic manifestation of the Bauschinger effect in such curves [23]. The HDI values were calculated using the LUR hysteresis loops, and the HDI values under different unloading strains are shown in Figure 4b. At a strain of 0.005, the HDI stresses of the two samples are comparable, with values of 559.4 MPa for UFG and 559.2 MPa for TW-UFG, indicating a negligible difference at the onset of plastic deformation. As the strain increases, HDI stress increases with rising unloading strain; however, the increase in HDI stress is greater for the TW-UFG specimens than for UFG specimens. At a strain of 0.15, the HDI stress of the TW-UFG sample reaches 743.1 MPa, which is approximately 29.6 MPa higher than that of the UFG sample. This progressively widening gap suggests that the presence of twin boundaries in the TW-UFG sample provides additional back stress, which enhances the strain hardening capability and contributes to the superior strength–ductility synergy observed at room temperature.

### 3.3. Mechanical Properties of Fine-Grained Strengthened ASS at Elevated Temperature

**Figure 5:**
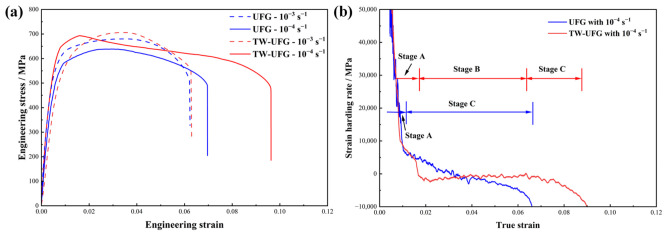
(**a**) Engineering stress–strain curves at 600 °C under strain rates of 10^−3^ s^−1^ and 10^−4^ s^−1^ and (**b**) work hardening rate curves under a strain rate of 10^−4^ s^−1^.

To investigate the effect of heterogeneous microstructures on the high-temperature mechanical properties of austenitic stainless steel, Figure 5 shows the engineering stress–strain curves of UFG and TW-UFG austenitic stainless steel at 600 °C under different strain rates. Compared to room-temperature tension, both samples exhibit a decrease in Young’s modulus, strength, and elongation at high temperature, consistent with previous research [24,25]. At various strain rates, the average yield strengths of both samples remain above ~550 MPa, indicating that grain-refinement strengthening still plays an important role. At a strain rate of 10^−3^ s^−1^, the total elongation after fracture of the UFG and TW-UFG samples during high-temperature tensile testing decreased by 65.0% and 67.3%, respectively, compared to room-temperature testing. However, as the strain rate decreased to 10^−4^ s^−1^, the elongation of the TW-UFG sample increased, reaching 1.38 times that of the UFG sample.

To analyze the differences in deformation behavior between the UFG and TW-UFG specimens during high-temperature tensile deformation at a strain rate of 10^−4^ s^−1^, the strain hardening rate (SHR) curves of the two sets of specimens were compared (Figure 5b). The SHR curve of the UFG specimen exhibited a two-stage decreasing trend: an initial sharp drop at low strain, followed by a slower decline until necking and fracture occurred. In contrast, the SHR curve of the TW-UFG specimen decreased below 0 MPa in stage A, then gradually increased toward positive values in stage B, and eventually declined again in stage C. The results indicate that the distinct behavior in stage B significantly enhanced the material’s strain hardening capacity and tensile ductility.

### 3.4. Microstructural Evolution During High-Temperature Tensile Deformation

**Figure 6:**
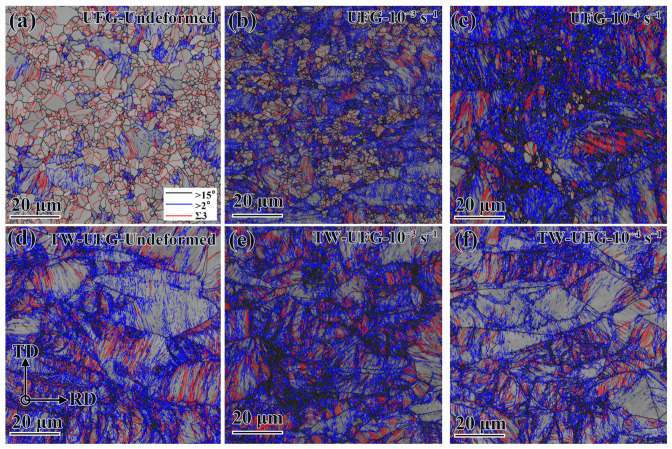
EBSD grain boundary maps of the (**a**–**c**) UFG and (**d**–**f**) TW-UFG specimens: (**a**,**d**) before tensile deformation and after tensile fracture at 600 °C at strain rates of (**b**,**e**) 10^−3^ s^−1^ and (**c**,**f**) 10^−4^ s^−1^, respectively. In (**b**,**c**,**e**,**f**), the observation surface is the RD-TD plane near the fracture surface. Grain boundary colors: the blue, black, and red lines represent low-angle (misorientation angle between 2° and 15°), high-angle (misorientation angle > 15°), and ∑3 twin boundaries (misorientation of 60° about a <111> axis, and the tolerance of misorientation angle for twin boundaries is 5°), respectively.

EBSD was used to statistically analyze the differences in the changes in grain boundary densities of different types between the UFG and TW-UFG specimens after high-temperature tensile fracture. Figure 6 shows the EBSD grain boundary maps at the high-temperature tensile fracture surfaces of the UFG and TW-UFG specimens under strain rates of 10^−3^ s^−1^ and 10^−4^ s^−1^. Table 2 summarizes the grain boundary density and average Kernel Average Misorientation (KAM) value at the high-temperature tensile fracture surface of the samples before stretching and under various strain conditions. The experimental results demonstrate that under strain rates of 10^−3^ s^−1^ and 10^−4^ s^−1^, the low-angle grain boundary density of the UFG specimens increased from 0.23 μm^−1^ to 1.44 μm^−1^ and 1.79 μm^−1^, respectively; in contrast, the density of high-angle grain boundaries remains relatively stable. The experimental results indicate that dislocation dynamic recovery continues under high-temperature tensile conditions, leading to a significant increase in the density of low-angle grain boundaries. While dynamic recrystallization has not yet initiated, the result in the ultrafine-grained microstructure is maintained, and no significant grain coarsening occurs.

According to the average Kernel Average Misorientation (KAM) of samples in Table 2, the UFG samples exhibited a uniform microstructure before tension, with a value of 0.45, and low-angle grain boundaries were predominantly concentrated in unrecrystallized grains. In contrast, TW-UFG samples underwent large deformation during processing, resulting in a higher average KAM value of 1.42. The average KAM values at the tensile fracture surfaces of the two groups of specimens showed opposite trends, and the average KAM value increased for the UFG specimen, while it decreased for the TW-UFG specimen. The increase in the average KAM indicates a greater strain gradient and more pronounced non-uniform deformation in the UFG specimen. The average KAM decrease for TW-UFG samples at a strain rate of 10^−4^ s^−1^ was greater than that at 10^−3^ s^−1^.

**Figure 7:**
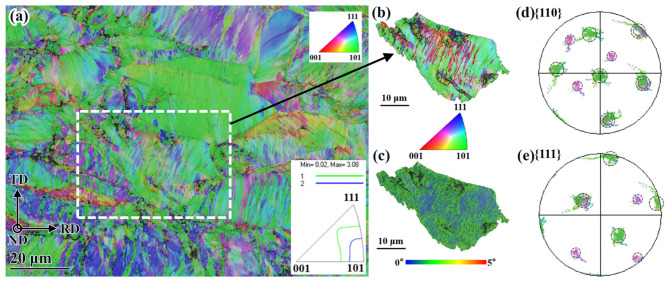
(**a**) EBSD IPF + IQ maps for the TW-UFG sample before tensile testing, (**b**) EBSD IPF + IQ + grain boundary maps corresponding to the location marked by a white frame in (**a**), (**c**) KAM map, and (**d**,**e**) pole figures showing twin relationship in (**b**). In (**b**), the black and red lines represent high-angle (misorientation angle > 15°) and ∑3 twin boundaries (misorientation of 60° about a <111> axis, and the tolerance of misorientation angle for twin boundaries is 5°), respectively.

To further investigate the influence of the heterogeneous microstructure in the TW-UFG samples on microstructural evolution during high-temperature tensile deformation, representative grains containing deformation twin bundles were selected for EBSD analysis. Figure 7a presents the orientation distribution map of the TW-UFG specimen before tensile deformation, revealing the typical texture characteristics of the pre-tension state. The untwinned deformed austenite regions exhibit a pronounced [101] orientation parallel to the normal direction (ND || [101]), as highlighted in green. It is well established that in face-centered cubic metals, the [111] orientation is a stable texture component during rolling [26]; however, the present [101]-oriented regions suggest that further rolling deformation would be less favorable for inducing deformation twinning. Representative grains marked with a white frame in Figure 7a, containing deformation twin bundles, were selected for further detailed EBSD analysis. As shown in Figure 7d, the deformation twin regions exhibit a 60° rotation about the <111> axis with respect to the parent austenite grains, consistent with the typical twin relationship in face-centered cubic structures. Consequently, three overlapping poles are observed in the {110} pole figure, while one overlapping pole appears in the {111} pole figure between the twin and the parent phase. A combined analysis of Figure 7b,c reveals color contrast variations at the twin boundaries, where the high KAM values indicate pronounced strain gradients and substantial GND accumulation.

**Figure 8:**
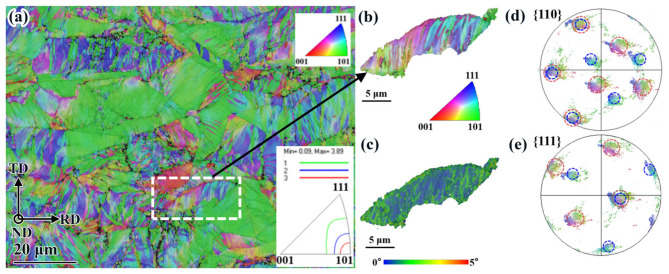
(**a**) EBSD IPF + IQ maps for the TW-UFG sample after tensile testing with 10^−4^ s^−1^ at 600 °C, (**b**) EBSD IPF + IQ + grain boundary maps corresponding to the location marked by a white frame in (**a**), (**c**) KAM map, and (**d**,**e**) pole figures showing twin relationship in (**b**). In (**b**), the black and red lines represent high-angle (misorientation angle > 15°) and ∑3 twin boundaries (misorientation of 60° about a <111> axis, and the tolerance of misorientation angle for twin boundaries is 5°), respectively.

In contrast to the pre-tension state, the [101] orientation is slightly enhanced after high-temperature tension (Figure 8a). As shown in Figure 8a,b, after high-temperature tensile deformation with 10^−4^ s^−1^, the deformed twin bundles remain parallel to the deformation bands. Meanwhile, the color contrast differences are reduced at the twin boundaries in the KAM maps, indicating that strain concentration at the twin boundaries has been alleviated. In the corresponding pole figures (Figure 8c,d), the grain boundaries retain the Σ3 coherent relationship; however, the pole distribution becomes more scattered compared to the undeformed state. This may be attributed to slight rotations of both the twin- and parent-phase lattices caused by dislocation motion during high-temperature deformation, leading to minor orientation divergence.

**Figure 9:**
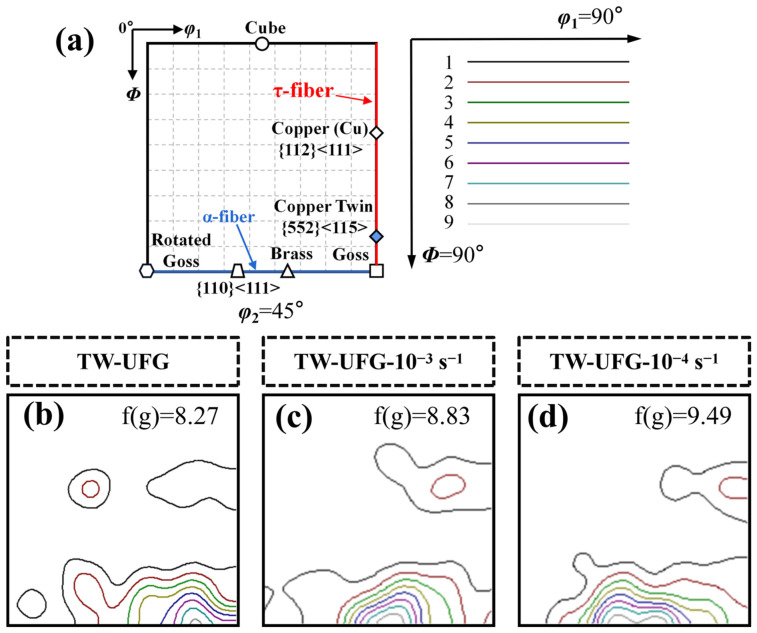
(**a**) The schematic illustration of the Euler space with important texture components for FCC; ODF maps (*φ*_2_ = 45°) for (**b**) the TW-UFG specimen prior to tensile testing; (**c**) fracture surface of the TW-UFG specimen tested at a strain rate of 10^−3^ s^−1^; and (**d**) fracture surface of the TW-UFG specimen tested at a strain rate of 10^−4^ s^−1^.

The texture evolution of the TW-UFG samples during high-temperature deformation was analyzed using the orientation distribution function (ODF, φ_2_ = 45° section) maps, as shown in Figure 9. During tensile deformation, grain orientations in the samples evolved toward stable {110}<111> and {112}<111> copper texture components at different strain rates. Meanwhile, the {552}<115> copper twin (Cu-T) texture at the fracture surface transformed into a Goss texture, with a reduction in Cu-T texture intensity compared to before deformation. Compared to other deformation textures, the Cu-T texture is closely associated with deformation twins; thus, its change in content can indirectly reflect the relative degree of twin participation [27,28]. Based on EBSD grain boundary statistics, the Σ3 grain boundary density in the undeformed TW-UFG specimen was 0.96 μm^−1^. After high-temperature tensile deformation with strain rates of 10^−3^ s^−1^ and 10^−4^ s^−1^, respectively, the Σ3 grain boundary densities near the fracture surfaces decreased to 0.82 μm^−1^ and 0.71 μm^−1^, which indicated the detwinning phenomenon of deformation twins in the TW-UFG specimen during high-temperature tensile deformation.

**Figure 10:**
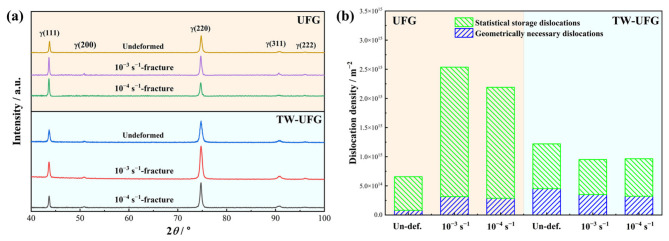
(**a**) XRD spectra and (**b**) average dislocation density at the fracture surfaces of UFG and TW-UFG specimens under a temperature of 600 °C and strain rates of 10^−3^ s^−1^ and 10^−4^ s^−1^, respectively.

The high-temperature mechanical properties of metallic specimens are closely related to the microstructural evolution during tensile deformation. Figure 10 shows the XRD diffraction patterns of two sets of specimens before high-temperature tension and at the fracture regions. The XRD results indicate that no deformation-induced martensite formed during high-temperature tensile deformation, as elevated temperatures increase the stacking fault energy of austenite, thereby suppressing the formation of deformation-induced martensite [29,30]. Using a modified Williamson–Hall method combined with XRD data, the total dislocation density was calculated, while the average GND density near the fracture region and prior to tensile deformation was determined using MATLAB^®^ toolbox-MTEX 6.1.0 based on the EBSD-KAM results. In the homogeneous UFG specimens, the total dislocation density increases significantly, primarily due to an order-of-magnitude rise in the density of statistically stored dislocations (SSDs), while the density of GNDs increases slightly. This indicates that deformation is dominated by homogeneous intragranular slip, with Taylor hardening arising from extensive dislocation tangling within the grains. In contrast, the TW-UFG specimens exhibit slight decreases in both total dislocation density and GND density, suggesting that dynamic recovery is activated at the elevated temperature.

**Figure 11:**
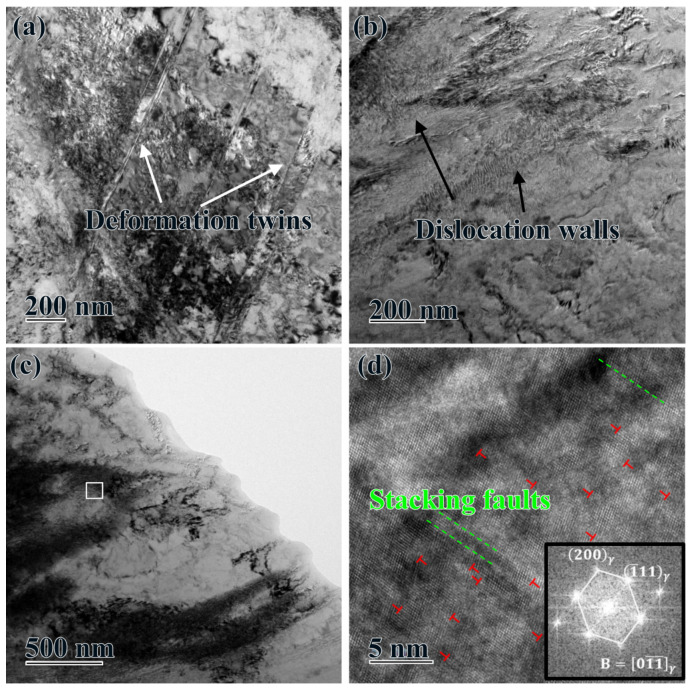
(**a**,**b**) TEM images of TW-UFG sample with the strain rate of 10^−4^ s^−1^ at 600 °C. (**d**) High-resolution TEM image taken from the white-boxed area in (**c**), with the fast Fourier transform spectrum shown as an inset. In (**d**) red symbols indicate dislocations, and the green dashed lines mark the stacking faults.

The microstructure near the fracture surface of the TW-UFG specimen under tensile deformation at 600 °C and a strain rate of 10^−4^ s^−1^ is shown in Figure 11. During tensile fracture, deformation twin bundles within grains and dislocations piled up at twin boundaries can still be observed (Figure 11a). Near grain boundaries, dislocation wall structures formed by recovery are visible (Figure 11b). The high-resolution transmission electron microscopy (HRTEM) analysis of the region marked by the white square in Figure 11c is shown as Figure 11d; the presence of stacking faults results in elongated diffraction streaks in the corresponding fast Fourier transform (FFT) pattern. HRTEM images reveal high-density stacking fault structures and dislocations, which promote work hardening during high-temperature tensile deformation.

### 3.5. High-Temperature Tensile Fracture Morphology

**Figure 12:**
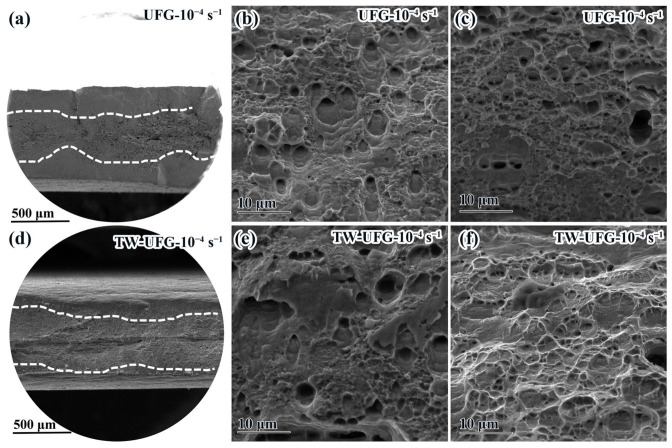
SEM fractographs of (**a**–**c**) UFG and (**d**–**f**) TW-UFG specimens after tensile fracture at 600 °C and 10^−4^ s^−1^. White dashed lines in (**a**,**d**) mark the boundary between the fibrous and radial regions.

SEM fractographs of the UFG and TW-UFG specimens after tensile testing at 600 °C and 10^−4^ s^−1^ are presented in Figure 12. The fracture morphology of both specimens exhibits typical ductile fracture characteristics, comprising fibrous zones in the central region and radiant zones near the edges. At low magnification (Figure 12a,d), the UFG specimen shows a relatively limited fibrous zone, whereas the TW-UFG specimen exhibits a notably larger fibrous zone accompanied by a smaller radiant zone. The enlarged fibrous zone in the TW-UFG specimen indicates a prolonged stage of stable crack growth, suggesting that more plastic strain was accommodated before the onset of rapid fracture. At higher magnification, the radiant zones of both specimens exhibit a relatively flat morphology with a small number of microvoids, as shown in Figure 12b,e. The fibrous zones of both specimens display typical ductile dimple morphology. The TW-UFG specimen exhibits larger and deeper dimples with a more uniform distribution across the fracture surface (Figure 12e) compared with the UFG specimen (Figure 12c). This phenomenon confirms that the microvoids underwent more extensive growth and coalescence during the deformation process, which is consistent with the enhanced uniform elongation of the TW-UFG specimen, as the formation of larger microvoids can absorb higher energy during ductile fracture.

## 4. Discussion

### 4.1. Twinning and Detwinning Behavior

The deformation mechanism of ASS is closely related to stacking fault energy (SFE), which increases with temperature [31,32,33]. The temperature dependence of SFE in ASSs has been extensively investigated through both experimental and theoretical approaches. Latanision and Ruff [34] measured the SFE temperature dependence in Fe-18.3Cr-10.7Ni and Fe-18.7Cr-15.9Ni (wt%) alloys over the temperature range of 27–327 °C, reporting values of |d*γ_SFE_*/d*T*| of 0.04 and 0.03 mJ m^−2^ K^−1^, respectively. Neding et al. [35] employed in situ high-energy X-ray diffraction combined with ab initio calculations over a broader temperature range of −45 to 450 °C for Fe-18Cr-15Ni, Fe-18Cr-17Ni, and Fe-21Cr-16Ni (wt%) alloys and consistently observed a linear increase in SFE with temperature, with a temperature dependence of approximately 0.05 mJ m^−2^ K^−1^. Molnár et al. [36] predicted a similar value of approximately 0.0483 mJ m^−2^ K^−1^ for 316L austenitic stainless steel (17.1Cr-10Ni-2Mo in wt%) over the temperature range of 300–800 K from ab initio calculations. Considering the very similar chemical composition and temperature range, the methodology of Molnár et al. [36] is considered applicable to determine the SFE of the present 17Cr-10Ni experimental steel. Based on the nearly linear temperature dependence of SFE established by the above studies [34,35,36], the SFE values of the present steel at 20 °C, 200 °C and 600 °C were obtained by linear extrapolation: 9.10 mJ m^−2^, 17.79 mJ m^−2^ and 36.87 mJ m^−2^, respectively. It should be noted that ab initio calculations yield the ideal SFE without considering the coherency strain energy associated with the volume contraction during the fcc-to-hcp transformation. To enable direct comparison with experimental data, a correction of +4 mJ m^−2^ was applied to the theoretical values, following the methodology of Pierce et al. [37]. Consequently, the corrected SFE values at 20 °C, 200 °C and 600 °C are 13.10 mJ m^−2^, 21.79 mJ m^−2^ and 40.87 mJ m^−2^, respectively.

This indicates that as the testing temperature increases, the deformation mechanism of stainless steel shifts from deformation-induced martensite transformation (low stacking fault energy ≤ 15 mJ/m^2^) to deformation-induced twinning dominated by higher stacking fault energy (~15–45 mJ/m^2^) [38]. At the initial stage of warm rolling deformation, dislocation slip is dominant. When local stress conditions reach a critical value, perfect dislocations in the perfect dislocation lattice decompose into 1/6<112> Shockley partial dislocations on {111} planes. These partial dislocations can glide continuously on adjacent {111} atomic planes, forming stacking faults. When multiple stacking faults stack in an orderly fashion, twins are formed, as shown in Figure 2c,f. At a tensile temperature of 600 °C, the SFE of the experimental steel reaches approximately 40.87 mJ/m^2^. Based on Byun’s Formula (2) for critical twinning shear stress (*τ_tw_*) [39]

(2) $$\tau_{tw}=\frac{2\gamma_{SFE}}{b_{p}}$$

In the equation, *γ_SFE_* is the stacking fault energy, and *b_p_* is the Burgers vector of the Shockley partial dislocation. Calculations show that the critical twinning shear stress *τ_tw_* during tensile deformation at 600 °C is approximately 551.9 MPa, while the shear stress corresponding to macroscopic yield strength is significantly lower than the twinning initiation threshold. Therefore, deformation twins are unlikely to form via stress-induced mechanisms during tensile deformation at 600 °C.

The synergistic effects of the multiaxial stress state and high-temperature environment govern the detwinning behavior during high-temperature tension. At 600 °C, no recrystallization was detected in the investigated steel; consequently, detwinning was influenced exclusively by dislocation–twin boundary interactions. For the relatively high SFE of materials, the formation of new deformation twins was suppressed during high-temperature tension. Instead, the observed reduction in twin boundary density is attributed to the progressive degradation of pre-existing deformation twins introduced during prior warm rolling. Two primary detwinning mechanisms operate under applied stress: (i) reverse twinning shear (π mode) and (ii) pseudo-reverse twinning shear (±π/3 mode) [40,41]. The π mode is activated when the resolved shear stress on a given twin system reaches the critical value *τ*_π_ = *γ*_isf_/*b_p_*, and the external stress orientation opposes the original twin shear direction, then inducing reverse fault slip at the twin boundary. The ±π/3 mode involves shear parallel to the twin plane but oriented at ±60° relative to the π-mode shear direction, and its critical resolved shear stress is typically approximately twice that of the π mode. Based on detwinning theory, the theoretical critical resolved shear stress for π-mode activation in this steel at 600 °C is ~275.9 MPa. For the heterogeneous TW-UFG specimen, the average Schmid factor is 0.44, and the measured average yield strength is 627.6 MPa at a strain rate of 10^−4^ s^−1^, so the average resolved shear stress is ~276.1 MPa—effectively matching the π-mode threshold. Under average stress conditions, the π-mode detwinning is expected to be preferentially activated, while the ±π/3 mode is unlikely to be activated on a macroscopic scale. However, the local stress concentrations, particularly at grain boundaries, twin boundaries, and dislocation pile-ups, can far exceed the average resolved shear stress and may potentially activate the ±π/3 mode at specific sites. As a result, following high-temperature tensile deformation at strain rates of 10^−3^ s^−1^ and 10^−4^ s^−1^, the Σ3 grain boundary density in the TW-UFG specimens decreased from an initial value of 0.96 μm^−1^ to 0.82 μm^−1^ and 0.71 μm^−1^, respectively.

### 4.2. The Relationship Between Heterostructures and High-Temperature Mechanical Properties

During room-temperature tension, the high strength of the heterogeneous TW-UFG sample, which is composed of nanoscale deformation twins and micrometer-scale coarse deformation austenite grains, is primarily attributed to grain-refinement strengthening and HDI stress hardening induced by the heterogeneous structure. During the processing stage, deformation-induced twinning produces bundles of nanoscale deformation twins that significantly refine austenite grains and contribute to grain boundary strengthening. Meanwhile, during the tensile deformation of the heterogeneous structure, high-density GNDs accumulate around the interfaces between different regions (hard zones and soft zones) [42]. The accumulation of these GNDs not only hinders dislocation slip at the interfaces but also generates long-range stress fields, leading to significant HDI stress hardening. In this study, the nanotwin region is regarded as the hard zone, whereas the untransformed micrometer-scale deformed austenite region serves as the soft zone. A high stress gradient forms around the interface of the heterogeneous structure, and the accumulated GNDs generate long-range stress fields that produce HDI stress in the direction opposite to dislocation motion [43]. The HDI stress hardening effect significantly enhances the material’s yield strength, elevating the strength of the soft region (unrefined micrometer-scale grains) to the level of nanoscale/ultrafine grains. With similar grain sizes, TW-UFG specimens with a heterogeneous structure exhibit higher HDI stress compared to UFG specimens with a homogeneous structure, resulting in superior strength–plasticity matching (as shown in Figure 4a).

The average yield strengths of the uniform UFG specimen at 600 °C under strain rates of 10^−3^ s^−1^ and 10^−4^ s^−1^ were 575.3 MPa and 558.9 MPa, respectively, indicating significant grain-refinement strengthening. EBSD grain boundary statistics revealed that the density of high-angle GBs near the tensile fracture remained nearly unchanged, suggesting that no obvious dynamic recrystallization occurred. However, the density of low-angle GBs increased significantly with both strain rates, indicating that a large number of dislocations rearranged into subgrain boundaries via climb and cross-slip. Therefore, it can be concluded that the dominant deformation mechanism in the high-temperature tensile process for the uniform UFG specimen is dislocation dynamic recovery [44]. In UFG samples, high-angle GBs exert dual effects on dislocations, including enhancing their storage while accelerating recovery. Dislocations are effectively absorbed by high-angle GBs, and the extremely short average dislocation glide distance in UFG samples promotes rapid annihilation of dislocations of opposite signs through climb at elevated temperatures. Meanwhile, numerous dislocations reorganize via dynamic recovery to form low-angle grain boundaries, which are stored within subgrain boundaries in low-energy, stable configurations, thus no longer effectively impeding subsequent dislocation motion and contributing minimally to work hardening. These factors collectively lead to the work hardening rate being offset by annihilation at very small strains. At a strain rate of 10^−4^ s^−1^, the work hardening rate dropped below zero at a true strain of only 0.034, resulting in the material entering the stage of plastic instability rapidly.

During high-temperature tension, twin boundary relaxation and detwinning become key features in the microstructural evolution of the TW-UFG ASS. At 600 °C, the ambient temperature increases the SFE, and the high density of GNDs stored near twin boundaries and random HAGBs obtains sufficient thermal energy to initiate dislocation climb and cross-slip behavior at interfaces. This thermally driven process enables the mutual annihilation of oppositely signed dislocations and the rearrangement of residual dislocations into lower-energy configurations, leading to the reduction in the local GND density at heterogeneous interfaces, and the HDI stress originating from GND pile-up is released. Apart from dislocation rearrangement induced by thermal activation, thermally promoted detwinning is another critical factor accounting for the attenuation of back stress. Previous studies have confirmed that interactions between dislocations and twin boundaries can promote detwinning within twin layers [45]. With the thermally activated conditions at 600 °C, the increased SFE enhances the mobility of Shockley partial dislocations [46]. Coherent twin boundaries gradually lose their coherent characteristics through stepwise annihilation and local migration. Once twin boundaries fail to act as rigid dislocation barriers, continuous dislocation pile-up is suppressed, and the long-range internal back stress induced by strain heterogeneity is accordingly diminished. As shown in Table 2, the average KAM values of the TW-UFG samples decrease after tension at 600 °C, suggesting a release of local stress concentrations at the room-temperature ex situ state. Subject to the EBSD step-size limitation, it can only be indirectly inferred from this microstructural characterization that the detwinning process reduces the local GND density, which may contribute to the relaxation of HDI stress during high-temperature deformation. At a strain rate of 10^−3^ s^−1^, the mechanical properties of TW-UFG specimens are comparable to those of uniform UFG specimens. However, with the strain rate decreased to 10^−4^ s^−1^, the elongation of TW-UFG specimens increases, demonstrating superior strength-plasticity balance compared to UFG specimens. During slow strain rate deformation, the Σ3 grain boundary density in the specimen decreases from 0.96 μm^−1^ to 0.71 μm^−1^, indicating the annihilation or migration of pre-existing twin boundaries. Detwinning effectively relieves local stress concentrations at twin boundaries, thereby suppressing premature crack initiation at these rigid interfaces. Concurrently, the removal of twin lamellae releases the partitioned space and provides an extended mean free path for subsequent dislocation motion. Meanwhile, extensive dynamic recovery promotes dislocation rearrangement, forming dislocation walls and high-density stacking faults (as shown in Figure 11c,d). Dislocation walls preferentially form near grain boundaries, subdividing original grains into finer subgrains. These subgrain boundaries achieve strain regulation through climb and slip, thereby reducing stress concentration [47]. As a result, the TW-UFG specimens reconstruct the work hardening rate during stage B, leading to a gradual rise in the work hardening curve, delaying plastic instability and enhancing elongation after fracture. The larger fibrous zone in the TW-UFG specimen reflects a prolonged stage of stable crack growth, indicating that the TW-UFG microstructure delays the onset of rapid fracture and allows more extensive plastic deformation before failure. In contrast, at the higher strain rate of 10^−3^ s^−1^, the time available for dynamic recovery is insufficient, resulting in incomplete dislocation rearrangement and limited stacking fault extension. Under this condition, detwinning primarily contributes to macroscopic softening, and the beneficial hardening effects are largely suppressed.

## 5. Conclusions

This study investigates the effects of heterogeneous microstructure and strain rate on the microstructural evolution and mechanical properties of ultrafine-grained ASSs during high-temperature tensile deformation. The main conclusions are as follows:

(1) At room temperature, the TW-UFG specimen with a heterogeneous structure exhibits higher tensile strength than the homogeneous UFG specimen due to HDI stress strengthening arising from interfaces between nanoscale twin bundles and micrometer-scale untransformed austenite grains, while maintaining good ductility.
(2) At 600 °C, the dominant deformation mechanism in the homogeneous UFG specimen is dislocation dynamic recovery, whereas in the heterogeneous TW-UFG specimen, it involves a coupled mechanism of detwinning and dislocation dynamic recovery.
(3) Under low strain rates (10^−4^ s^−1^), the TW-UFG specimens show favorable strength–plasticity synergy over UFG specimens, attributed to detwinning and dynamic recovery. Detwinning relieves stress concentrations and removes boundary partitioning, while dislocation walls and stacking faults from dynamic recovery sustain work hardening and delay instability.

## Figures and Tables

**Table 1:** Chemical composition of 17Cr-10Ni austenitic stainless steel (wt.%).

C	Cr	Ni	Mn	Mo	Si	P	S	Fe
0.02	16.83	10.38	1.435	2.07	0.554	0.031	0.005	Bal.

**Table 2:** The grain boundary density at different misorientation angles and average KAM values of UFG and TW-UFG specimens fractured via tensile deformation at 600 °C with strain rates of 10^−3^ s^−1^ and 10^−4^ s^−1^, respectively.

Samples	Grain Boundary Density, Length/Area, μm^−1^			Average KAM Value
	2°~5°	5°~15°	>15°	
UFG	0.10	0.13	1.19	0.43
UFG-10^−3^ s^−1^	0.84	0.60	1.14	1.59
UFG-10^−4^ s^−1^	0.81	0.98	1.17	1.24
TW-UFG	0.85	0.77	1.53	1.42
TW-UFG-10^−3^ s^−1^	0.79	0.74	1.45	1.29
TW-UFG-10^−4^ s^−1^	0.54	0.61	1.29	1.15

## Data Availability

The original contributions presented in this study are included in the article. Further inquiries can be directed to the corresponding author.
